# Recognizing and Responding to Child Maltreatment: Strategies to Apply When Delivering Family-Based Treatment for Eating Disorders

**DOI:** 10.3389/fpsyt.2020.00678

**Published:** 2020-07-10

**Authors:** Melissa Kimber, Andrea Gonzalez, Harriet L. MacMillan

**Affiliations:** ^1^ Offord Centre for Child Studies, Department of Psychiatry and Behavioural Neurosciences, McMaster University, Hamilton, ON, Canada; ^2^ Department of Pediatrics, McMaster University, Hamilton, ON, Canada

**Keywords:** child maltreatment, eating disorders, children, adolescents, intervention, mandatory reporting

## Abstract

Child maltreatment encompasses a constellation of adverse parental behaviors that include physical, sexual, or emotional abuse, physical or emotional neglect, as well as exposure to violence between parents. A growing body of literature indicates that exposure to child maltreatment is a significant risk factor for the development and maintenance of eating disorders (EDs) and that practitioners experience challenges related to recognizing and responding to various forms of child maltreatment in their practice. Parent-child interactions signifying possible child maltreatment can be subtle; furthermore, the emotional and behavioral symptoms associated with an ED can overlap with those linked with child maltreatment, making it difficult for practitioners to distinguish whether children’s symptoms are attributable to underlying psychopathology versus exposure to child maltreatment. This challenge can be further complicated in the context of delivering family-based treatment (FBT); FBT reaffirms that there is no single cause of EDs and asserts the leadership role of parents in their child’s recovery process—both of which may lead practitioners to inadvertently miss indicators of child maltreatment. In this article, we provide an overview of the evidence linking child maltreatment to EDs among children and adolescents, as well as evidence-informed strategies for practitioners to safely recognize and respond to suspected child maltreatment when delivering FBT to children and adolescents in their practice.

## Introduction

Maltreatment in childhood or adolescence is a non-specific risk factor for a substantial proportion of mental disorders detailed in the Diagnostic and Statistical Manual of Mental Disorders (DSM);(1–3) this includes eating disorders (EDs) (3, 4). Generally speaking, child maltreatment includes physical, sexual, or emotional abuse, as well as physical and/or emotional neglect by a parent or caregiver (hereafter referred to as “caregiver”) toward a child (i.e., under 18 years of age) which results in actual or potential physical or emotional harm to the child. In addition, children’s exposure to intimate partner violence (IPV) between adults is increasingly considered a form of child maltreatment; (5) its negative impacts are similar to other forms of child abuse and neglect (3, 4, 6). **Table 1** provides a definition for each type of child maltreatment, as well as examples of adverse caregiver behaviors that are characteristic of each child maltreatment type. Importantly, rates of exposure to child maltreatment among individuals with an ED are high relative to population norms, as well as individuals with other types of psychopathology (8, 9). In addition, individuals with an ED and a history of maltreatment tend to have earlier ED onset, higher rates of psychiatric comorbidity, a greater frequency of suicidal ideation and attempts, as well as a more severe course of eating-related pathology compared to their non-maltreated peers (8–10). The objectives of this paper are to provide an overview of the evidence linking child maltreatment to EDs among children and adolescents. Second, we discuss how the strict adherence to the principles of family-based treatment (FBT) for EDs has the potential to interfere with a practitioner’s ability to readily recognize and respond to any historical or ongoing child maltreatment exposure. We follow this discussion by providing evidence-informed strategies for practitioners to safely recognize and respond to suspected or disclosed child maltreatment when implementing FBT with children, adolescents, and their families in practice.

**Table 1:** Types and Examples of Child Maltreatment (7).

Child Maltreatment Sub-type	Definition	Example Adverse Parenting Behaviors
Physical abuse	Includes the use of physical force, a tool or apparatus to physically harm or control a child or adolescent. Physical abuse has also occurred when a parent or caregiver commits an act that can or does result in physical injury to the child or adolescent, including red marks, cuts, welts, bruises, muscle sprains, head injury, and/or broken bones.	Hitting Kicking Shaking Throwing Poisoning Burning or scalding Drowning Suffocating Deliberately inducing illness *via* intentional exposure to known communicable diseases or viruses.
Sexual abuse	Includes forcing or enticing a child to take part in sexual activities, whether or not the child is aware of what is happening to them. The activities may involve penetrative and non-penetrative acts and non-contact activities.	Inappropriate fondling of the child or touching of any kind. Vaginal, anal, or oral rape, or attempted rape. Making the child read, watch, hear or participate in sexual acts or pornography. Talking about sex, sexual activity, and sexual acts with a child that is outside the scope of developmentally appropriate discussions of sex and sexuality.
Emotional abuse	Used interchangeably with psychological abuse, this maltreatment involves a non-physical, repeated pattern of caregiver behavior that is likely to be interpreted by the child that they are disliked, unwanted, unloved or rejected by the caregiver and which undermines the child’s development, well-being and socialization. If severe enough, a single incident by a caregiver could be considered emotionally abusive if deemed emotionally harmful or potentially harmful for a child.	Spurning the child (belittling, denigrating, ridiculing, humiliating in public). Terrorizing the child (e.g., placing in unpredictable or chaotic circumstances, placing the child in dangerous situations, etc.). Isolating the child (e.g., confining the child within a room, structure or environment for a non-medically necessary reason; or restricting social interaction within the community, etc). Exploiting or corrupting the child’s behavior and development (e.g., modeling, permitting or encouraging antisocial behavior, purposefully restricting the child’s cognitive development)
Emotional neglect	Used interchangeably with psychological neglect, emotional neglect involves caregivers repeated acts of omission and which place their child at risk of emotional harm; including a caregiver’s repeated failure to notice, attend to, or respond appropriately to a child or adolescent’s feelings.	Denying affection when warranted for the child or requested by the child. Demonstrating coldness or little-to-no warmth toward the child. Ignoring or refusal to engage with the child when demonstrating certain emotions (e.g., anger, sadness, excitement, etc.). Providing no praise when warranted
Physical neglect	Refers to a caregiver or parent’s inability, refusal, or failure to provide their child with the resources needed for healthy physical development, as well as the level of monitoring and supervision needed to keep the child physically safe from harm or potential harm that is within the control of the caregiver.	Failure or inability to provide: Adequate food for the child’s growth and development. Clothing adequate for the weather. Necessary medical or dental care for the child’s development and continued well-being. Adequate shelter for the child.
Child exposure to intimate partner violence	Refers to any child or adolescent’s exposure to, witnessing or awareness of any incident of violent or threatening behavior between adults who are or have been intimate partners.	Failure or inability to prevent one’s child from witnessing or having an awareness of: Physical violence (e.g., slapping, kicking, punching, beating, etc.) between adult caregivers. Emotional abuse (e.g., belittling, degradation, humiliation, etc.) between adult caregivers. Consensual and/or non-consensual sexual behavior (touching, petting, viewing of pornographic material, intercourse, rape, etc.), between adult caregivers. Financial control or abuse of the other caregiver/parent for purposes of socially controlling or harming (e.g., precluding one parent/caregiver from having access to household finances, spending uncharacteristically large sums of money to punish the other parent/caregiver; etc.).

Adapted from: National Institute for Health and Care Excellence (7).

High rates of a history of child maltreatment have been found among adults with all ED diagnoses, including anorexia nervosa restricting and binge-purge subtypes.(8–10). Most studies focus on female adult samples. In addition, studies vary in their approach to measuring child maltreatment (11). Recent reviews and meta-analyses (8–11) indicate that the prevalence of any form of child maltreatment ranges from 21 to 66%, though much of the literature has focused on physical, sexual, and emotional abuse (8–11). To our knowledge, there is no published study detailing the prevalence of children’s exposure to IPV among youth or adults diagnosed with EDs. However, a review of studies based on adult retrospective self-reports indicates that conservatively, at least 10–20% of youth are exposed to IPV annually; high rates of this exposure have been replicated in a national sample of US youth (12–15). Lastly, it is important for practitioners to be aware that different forms of child maltreatment tend to co-occur (16, 17) and exposure to more than one form of child maltreatment is strongly associated with disordered eating behavior in adults (8) and adolescents (18).

Evidence suggests that the inability to express emotion in healthy and adaptive ways may be the mechanism linking child maltreatment exposure and the development of an ED. Caregivers who maltreat their children show less positive emotion (i.e., joy and warmth), more negative emotion (e.g., anger and sadness), and have less favorable perceptions of emotional expression than their non-maltreating peers. Each of these factors is associated with emotion processing challenges in their offspring, as well as the onset, severity, and duration of child mental health problems and successful intervention (19). Children who experience inconsistent, harsh, dismissive, or overinvolved caregiver responses to their emotions have been shown to demonstrate deficits in emotion processing, including recognizing, understanding (e.g., labeling), and expressing emotion. More specifically, when these caregiver responses occur, the emotional experience of the child is ignored, minimized, or incongruent with their expectation. If this emotional incongruence happens frequently or repeatedly, this can contribute to the internalization of negative beliefs toward emotional expression. Such internalizations may alter the child’s cognitive processing of emotion information and lead to the use of maladaptive strategies (e.g., emotional suppression) (20–22) to process emotion, as well as the subsequent development of behavioral and psychological symptoms (e.g., purging, restriction, binge eating, excessive exercise, etc.), to manage their emotion states (23–26). Though this explanatory framework is a promising line of research, additional longitudinal studies capable of disentangling the influence of maltreatment exposure on maladaptive emotion processing and ED development, persistence and intervention are needed.

## Recognizing Child Maltreatment Within the Therapeutic Stance of FBT

A recent qualitative meta-synthesis highlighted that healthcare and social service providers experience challenges related to recognizing and responding to child maltreatment in their practice (27). Emerging work suggests that this is also the case among ED specialists (28). There are several potential implications of this practice challenge. First, FBT continues to be the first-line intervention for youth diagnosed with anorexia nervosa and bulimia nervosa; (29, 30) additional evaluations about the tenability of the intervention for treating binge ED, as well as other-specified feeding and EDs are being explored (31–33). As a behaviorally-based intervention, FBT involves several key processes and principles that could complicate practitioners’ ability to effectively and efficiently recognize and respond to child maltreatment. These include a non-determinant view of the illness, a non-authoritarian therapeutic stance, empowerment of caregivers to facilitate the recovery process, as well as an initial prioritization on nourishment and symptom interruption (34–36). In the context of FBT, an unequivocal belief that caregivers are a positive resource to their child’s recovery has the potential to negate the identification of adverse caregiver behavior in the context of a child’s physical and psychological vulnerability, as well as magnify the impacts of any ongoing child maltreatment (28). Second, a non-determinant view of the illness may implicitly suggest to practitioners that attending to the relational patterns in the family system is secondary to the emphasis on symptom interruption. However, it is important to note that chronic, cumulative exposure to child maltreatment may lead to enduring vulnerability to ED sequelae. In particular, exposure to child maltreatment has been implicated in suboptimal functioning of the system responsible for neuroendocrine stress regulation—the hypothalamic-pituitary-adrenal (HPA) axis. Emerging evidence indicates that altered HPA-axis functioning has the potential to mediate the onset, severity, and persistence of mental health symptoms, (37, 38) including ED pathology (39–42). In addition, recent studies suggest the possibility of protracted psychiatric vulnerability *via* changes to the HPA-axis among individuals with an ED and a child maltreatment history given the effects of ED symptomology (e.g., starvation, binge-purge cycles) on the neuroendocrine response system (43, 44). Thus, recognizing and halting any ongoing child maltreatment exposure is critical to short and long-term recovery from an ED.

**Figure 1:**
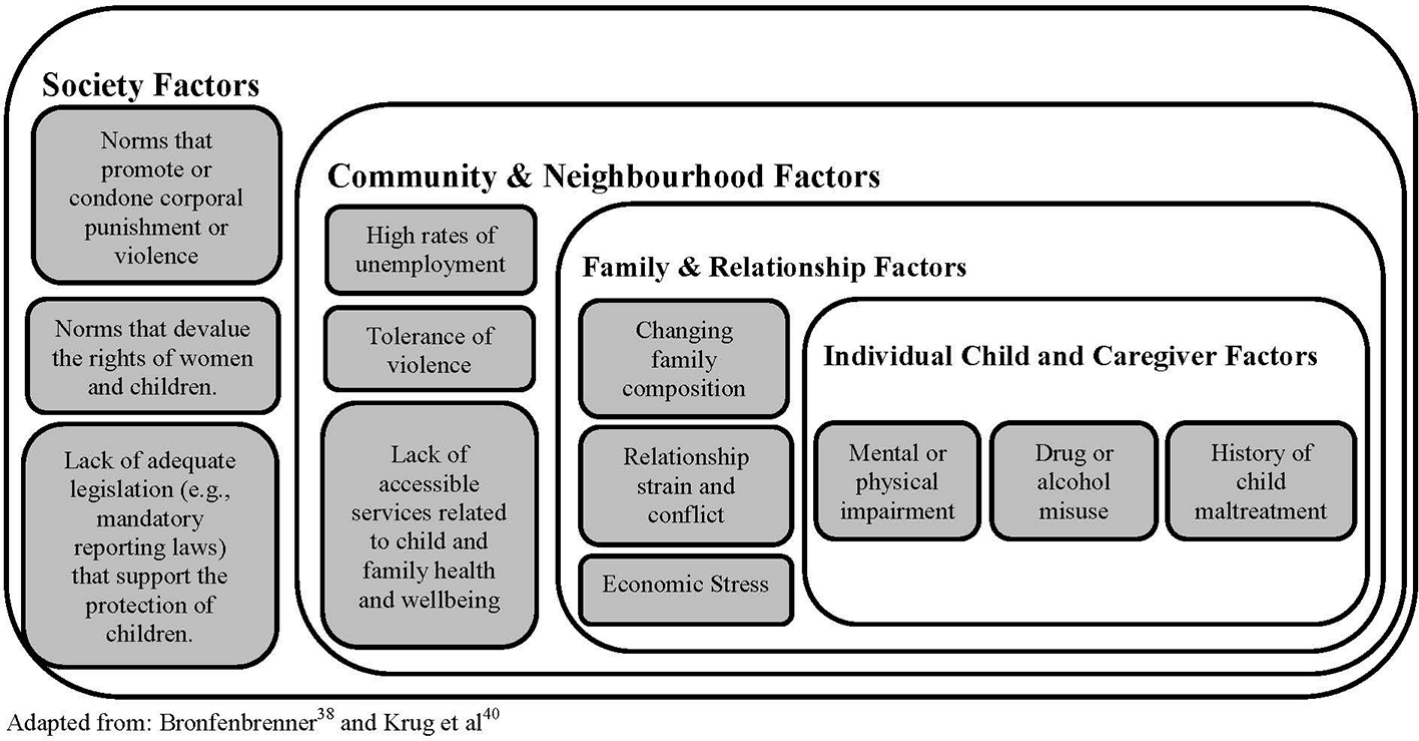
Adapted Ecological Model for Child Maltreatment—Risk Factors.

Safe recognition and response to child maltreatment refers to the practitioner’s use of assessment and therapeutic strategies that limit the possibility of additional harm related to child maltreatment exposure. There are two key considerations relevant to the safe recognition of child maltreatment: (a) engaging in an ongoing assessment of child maltreatment risk factors; and (b) being attuned to the interactions between a child and their caregiver. The literature cites several common sources of risk for child maltreatment, which can be understood within an adapted version of Bronfenbrenner’s (45, 46) ecological model of human health and development. Framed within this model, child maltreatment exposure can be considered a consequence of multiple risk factors at the individual, family, community, and societal-level and which interact to create the conditions in which child maltreatment has a stronger likelihood of occurring (45, 47, 48). An overview of the risk factors associated with child maltreatment exposure are provided in **Figure 1**. Briefly, the experience of multiple and chronic stressors within and outside the family system is associated with child maltreatment exposure. For example, stress related to caregiver unemployment may be compounded by a child or caregiver’s mental health challenges, which can place caregivers at greater risk of maltreating their children. Changes in family demographics (e.g., pregnancy, martial separation, etc.), as well as chronic family conflict (e.g., custody disputes, disparate caregiving practices, etc.), are contexts in which children are at greater risk of being maltreated. In addition, tolerance for discrimination on the basis of gender, racial, or ethnic identity within one’s community, as well as societal social and cultural norms that condone the use of violence can all contribute to the risk of child maltreatment (47–52).

Importantly, all forms of child maltreatment can lack overt indicators of their occurrence. In addition, children and youth with and without maltreatment exposure and who are presenting for ED intervention can exhibit similar emotional and behavioral symptoms. For example, the “alerting features” for child maltreatment listed in the National Institute for Health and Care Excellence (NICE) Guidelines (7) (pg. 9) overlap with the symptoms associated with ED psychopathology (e.g., significant changes in mood, etc.). For these reasons, initial and ongoing assessment of the relational patterns within the family and based on information from multiple family members is crucial for safe and efficient recognition and response. This approach is consistent with those detailed in the FBT manuals, which indicate that a comprehensive assessment that consists of an interview with the child, the caregiver(s), a medical evaluation and the administration of standardized questionnaires is critical. The assessment should include a review of the child’s ED symptoms and their onset, family dynamics, as well as the impacts of the illness on the youth’s short and long-term goals for academics, peer, and family relationships (34, 36).

The FBT manual suggests that practitioners should meet with caregivers separately from the youth. In addition, some time spent with each caregiver alone is important for ensuring that any inquiry related to child maltreatment—and in particular, exposure to IPV—is done safely. The two most common forms of IPV are situational couple violence and intimate terrorism; the former is rooted in couple conflict that escalates to violence and the latter refers to an interactional pattern whereby one partner explicitly attempts to exert general, long-term control, over the other partner; this is known as coercive control (53). In the context of intimate terrorism, coercive control tends to encompass three key characteristics: (a) the use of coercion *via* demand or threat; (b) the abuser’s ability and willingness to follow through on threats; and (c) the surveillance of a victim’s activity with the intention to make good on the threats (54). In the context of an FBT assessment, initial interviews with caregivers could raise issues of conflict related to disparate caregiving practices, financial challenges, disagreements about their child’s diagnosis, as well as the perceived need for intervention, and other family-related challenges, including violence. Given this information, as well as the possibility that violence could intensify in the context of psychosocial stress related to their child’s serious illness, (55, 56) it is critical that each caregiver be provided an opportunity to meet with the FBT therapist independently from their partner. In doing so, FBT practitioners can prioritize the safety of any caregiver who is currently experiencing IPV, as well as safely inquire about child exposure to the IPV, and gain a clearer understanding of the dynamics of the caregiver-dyad, which is critical to the FBT process.

### Principles for Safely Recognizing Child Maltreatment in FBT

The principles for safely recognizing child maltreatment in the context of delivering FBT align with the general principles for providing trauma-informed care (TIC). TIC refers to mental health care that is strengths-based and which explicitly seeks to minimize the possibility of iatrogenic effects related to the therapeutic process (57, 58). TIC recognizes that exposure to trauma, including child maltreatment, is widespread in the population and that trauma is a strong antecedent of mental health challenges. From a practice perspective, TIC involves the key elements of: (a) ensuring patient collaboration and autonomy; and (b) a commitment to inquiring about and responding to experiences of trauma sensitively and in a way that prioritizes the patient’s physical *and* emotional safety (58). The last decade has seen significant advocacy and discussion related to TIC and though there remains little empirical evidence related to how best to provide TIC in mental health settings, (59) many of its principles parallel recommendations for good clinical practice. Aligning with the key elements of TIC, there are three key principles for safely recognizing child maltreatment in the context of delivering FBT. First, universal screening for child maltreatment is not recommended at any point during FBT. There is no evidence that universal screening for child maltreatment leads to reduced harm, reduced exposure, or optimal outcomes among those who have experienced child maltreatment (60–63). Second, an explicit discussion about the limits of confidentiality with each family member is needed. During the initial assessment, the family should be advised about the limits of confidentiality, jointly. An additional review about the limits of confidentiality should be completed with each family member who participates in an individual interview as part of the assessment process; special attention should be paid to ensuring that youth understand the concept of safety and the limits of confidentiality when issues of safety arise. Third, practitioners should implement a phased inquiry approach. Specifically, practitioner questions about potential child maltreatment exposure should begin with questions focused on the presenting concern (i.e., disordered eating and the onset of symptoms) and then proceed to ask broader questions about well-being and safety. For example, after taking a history of ED symptoms and behaviors, the practitioner might ask: “how does everyone get along at meal times,” and then “what about outside of meal times?” These questions might be followed by, “what happens when someone gets upset or angry,” and then, “what’s the worst thing that has happened?” If the practitioner is considering physical abuse and notices an overt sign for inquiry (e.g., large bruise on a child’s leg), in the individual interview with the child, they may say something like, “I see that you have a bruise on your leg. Tell me about that, how did it happen?” There is no evidence to support routine screening for child maltreatment—in other words, asking everyone a standardized set of questions about child maltreatment exposure, irrespective of presenting signs and symptoms is not supported by evidence. A phased inquiry is the safest and most robust approach for recognizing child maltreatment when signs and symptoms suggest possible exposure.

### Opportunities for Ongoing, Phased Inquiry During FBT

Processes embedded within the delivery of FBT provide additional opportunities for safe, ongoing, inquiry into historical, or ongoing maltreatment. These processes include the discussion of the caregiver dyad’s efforts to support their child to interrupt ED symptoms, as well as sibling(s’) efforts to support the patient. Practitioners can continue to monitor family dynamics with input from multiple family members. In addition, as detailed in the FBT manuals, each session begins with the practitioner weighing the youth, which is a process that is about 10 min in duration and completed separately from the caregivers and siblings (34, 36). During this time, practitioners can check in with the youth individually. In line with FBT, practitioners can initiate the inquiry by asking youth, during their weigh-in, about how meals and snacks have been going since the last session, as well as asking about any instances of ED behavior (e.g., restriction, exercise, binging, purging, etc.),. This can be followed with open-ended questions that elicit the youth’s recall of their caregiver’s reactions and interactions with the adolescent (e.g., after you came back from exercising, what happened)? that relate to ED symptomology and the FBT model more generally. Potential “signals” for phased inquiry include the sustainment or worsening of ED or comorbid mental health symptoms, misalignment in reports of progress (or lack thereof) between caregivers, youth and/or their siblings, as well as sudden worsening of the youth’s or caregiver’s mood, willingness to engage in treatment, as well as their interactions with each other.

## Responding When There is a Suspicion or Disclosure of Child Maltreatment in the Context of FBT

Fears of being reported to child protection authorities, as well as the potential outcome of a report, can influence a family’s ongoing attendance at sessions. It can also influence the information that youth and caregivers share with their practitioners (64). Importantly, in jurisdictions with mandatory reporting, a suspicion of maltreatment meets the threshold for a report to child protection authorities (although the definition of maltreatment can vary) (65, 66). For this reason, the reporting of suspicions and disclosures of child maltreatment constitute a principal (and challenging) component of the response process. The NICE guidelines offer important information regarding the distinction between “considering” and “suspecting” child maltreatment (7). To “consider” child maltreatment means that “child maltreatment is one possible explanation” (among others) for the sign or symptom (7). To “suspect” child maltreatment means that the practitioner has a serious level of concern about the possibility of child maltreatment exposure (7). It is not the responsibility of FBT practitioners to obtain proof of child maltreatment exposure and it is important that practitioner’s inquiry about signs and symptoms, as well as their responses, do not interfere with the role of child protection professionals. A practitioner’s suspicion of maltreatment is justified when the explanation for a sign or symptom is unreasonable or unsuitable. This would mean that the explanation is implausible, inconsistent, or insufficient relative to the child’s typical activities, presentation, medical condition, or their developmental trajectory; in these instances, the explanation for the sign or symptom may differ between caregivers, between accounts over time, and/or differs between a child and their caregiver. Importantly, under no circumstances should caregiver preferences for culturally-based practices, rituals or behavior justify emotional or physical harm toward a child. To this end, it is essential for practitioners to be familiar with the legislative requirements concerning the threshold of suspicion and the protection of youth. In addition, it is helpful for practitioners to be aware of, and utilize strategies that can be facilitative of more positive response and reporting experiences. These include empathic responding, appropriate documentation procedures, and ensuring the provision of ongoing support.

### Empathic Responding and Mandatory Reporting in FBT

When child maltreatment is suspected or disclosed, it is important to respond to the information that has been shared in a respectful and compassionate manner. Acknowledging that it can be difficult to talk about these experiences and thanking clients for sharing the information with you is important for respecting the vulnerability of their position and the trust they have placed in you as their practitioner. When a suspicion of child maltreatment has arisen or a disclosure of child maltreatment has been made, it is important to directly communicate your professional concern about what has been shared. Second, the youth and caregiver should be reminded about the limits of confidentiality. Upon doing so, the practitioner can then share that their professional concern warrants a report to the child protection authorities, whose role is to support families. Only in instances where the practitioner is concerned that the caregiver may flee with the youth or when there is an imminent safety risk, should the practitioner withhold their plan to report to child protection professionals from the caregiver or the child. As much as possible and where safe to do so, the non-offending caregiver’s participation in the practitioner’s report to child protection services (CPS) should be considered and implemented. The youth and their caregiver should be informed about the CPS’ potential responses to the report; the practitioner should refrain from making any definitive statements about what will occur. Letting the youth or caregiver know that CPS responses are family-specific, is important. The youth and caregiver can be advised that the practitioner will ask CPS about what they plan to do and that this will be shared where possible with the family. Practitioner transparency about the process, when appropriate and safe to do so, can demonstrate the practitioner’s ongoing commitment to supporting the youth and caregiver in the process.

When making the report to CPS, the practitioner should communicate to CPS any and all information that is relevant to their concern about suspected or disclosed child maltreatment; details that are not relevant should not be shared. It is important that an interpretation of what has reportedly occurred not be offered, unless it is within the practitioner’s scope of practice (e.g., the individual is an expert in child maltreatment impact). In addition, it is important for the practitioner to clarify with CPS directly what they anticipate for their ongoing role. For example, the practitioner should communicate whether the family will continue to be seen for ED treatment or whether further sessions will be put on hold until after CPS has completed an assessment. In addition, it is possible that a report to CPS will result in the family’s termination of the relationship with the FBT practitioner. If a family indicates that they will not return for FBT, as much as possible, providers should offer to connect the youth and caregiver to a new provider whose scope of practice includes ED intervention. The plan should be communicated to the youth and caregiver and the CPS professional. Any concerns that the practitioner has about the delay in treatment for the ED can be made explicit to CPS. It is important for the practitioner to carefully document interactions with the youth, caregiver, and CPS professionals throughout the process. Good documentation practices include: (a) recording verbatim statements by the youth or caregiver; (b) noting discrepancies in the youth’s or caregiver’s account, if any, and without interpretation; (c) recording a detailed description of the youth’s emotional state, behavior, symptoms, or injuries; (d) a note that the call to CPS was made; (e) a note as to whether or not a conversation with the caregiver and/or child about contacting CPS occurred before and/or after the call (if appropriate); and (f) a note about what CPS communicated as potential next steps. Additional guidance for making a report to CPS is available elsewhere (27, 64, 67).

### Providing Support Alongside CPS

It is important for ED specialists to have familiarity with their local CPS. To date, there are no guidelines as to whether or not FBT should continue when child maltreatment is suspected or disclosed. Plans for ongoing FBT that involves an offending or non-offending caregiver should be informed through collaboration with CPS. If working with a caregiver raises safety concerns or if the CPS professional indicates that caregiver involvement is not possible, there are options that can be discussed with CPS. These include reconstituting the family for the purposes of FBT (e.g., having an aunt/uncle participate in the therapy) or implementing an individual-based psychotherapeutic approach with the youth. For example, Adolescent Focused Psychotherapy for Anorexia Nervosa (AFT-AN) (68) is a developmentally-oriented, individual-based model of psychotherapy that focuses on supporting the youth to address negative affective states that are assumed to be driving ED behavior. Compared to FBT, AFT-AN has been shown to be similarly effective in producing full-remission of ED symptoms among youth with anorexia at end-of-treatment (69). Similarly, Cognitive Behavioral Therapy for Eating Disorders (CBT-ED) (70) or CBT Enhanced (CBT-E) (71–74) may be appropriate for youth with anorexia, bulimia, or other-specified food and EDs where caregiver involvement is not appropriate or possible. Importantly, AFT-AN, CBT-ED, and CBT-E have not been evaluated among samples of youth with identified maltreatment exposure, but are listed in guidelines, systematic reviews and meta-analyses as possible interventions for the treatment of youth EDs when FBT is not available, appropriate, or when FBT is contraindicated (29, 30, 75, 76).

In cases where a youth has disclosed exposure to child maltreatment or when exposure has been confirmed by CPS professionals, it is recommended that the FBT practitioner seek consultation with a trauma or child maltreatment expert. A child maltreatment impact assessment may determine that FBT should cease or that adjunctive intervention for trauma-related symptoms is needed. For example, for youth who are exhibiting posttraumatic stress disorder symptoms, a referral to services offering cognitive behavioral therapy (CBT) with a trauma focus (TF-CBT) is appropriate; (77, 78) in particular, the TF-CBT model detailed by Cohen, Mannarino, and Deblinger (79–81). Similarly, for youth up to the age of 12 who have been exposed to physical abuse or neglect and are exhibiting externalizing symptoms, the family can be referred to parent-child interaction therapy (PCIT) (77, 82). The literature also indicates that in the absence of an available intervention, the provision of psychoeducation about trauma and its associated responses can be an important initial step in addressing trauma-related symptoms (83). Importantly, there is no evidence to support universal screening for exposure to adverse childhood experiences (ACES)—which includes exposure to child maltreatment (84–86). Recent papers report insufficient evidence that ACES screening yields any health benefits for individuals with a history of exposure to child maltreatment (84). In addition, the World Health Organization (87), recommends against universal screening for child maltreatment in the context of mental health and developmental assessments for children and youth. As with the continuation of FBT, any screening, ongoing care or referrals provided to the youth or caregiver for trauma-related symptoms should be clearly documented and made in collaboration with a trauma expert and CPS professionals.

## Conclusions

There is very little information on how exposure to various types of child maltreatment influences the provision of mental health interventions, including FBT for EDs. ED practitioners need to consider child maltreatment exposure in the assessment of youth; safe inquires and responses build upon the principles of TIC, as well as the communication and psychotherapeutic skills that are already central to their practice. Recognition and response to suspected child maltreatment within the context of FBT involves a clear, developmentally appropriate discussion about the limits of confidentiality at the outset of the therapeutic process. This is followed by a phased inquiry that starts with the presenting concern and which elicits reasonable explanations for signs and symptoms. General questions about interpersonal relationships should be included in the assessment. In those regions where mandatory reporting laws are in place, when practitioners suspect child maltreatment, a report to CPS should be made. Safe responses to suspected maltreatment begin with listening and conveying an emphasis on keeping the child safe. This should be followed by the report to CPS (where required) and the development of a plan for ongoing care related to the youth’s mental health, which may or may not include FBT. Critically, FBT practitioners are not required to be experts in the recognition and response to child maltreatment; they are also not required to be experts in the assessment of trauma symptoms or the delivery of trauma-focused care. However, they do have a professional and ethical responsibility to do what they can to ensure the safety and well-being of the children and adolescents for whom they are providing FBT.

## Author Contributions

MK wrote the first draft of the manuscript. AG and HM contributed to the conception, editing, and research for the manuscript. All authors contributed to the article and approved the submitted version.

## Funding

AG is supported by a Canadian Institutes of Health Research Tier 2 Canada Research Chair and an Ontario Ministry of Research, Innovation and Science Early Researcher Award. HM is supported by the Chedoke Health Chair in Child Psychiatry at McMaster University.

## Conflict of Interest

MK and HM report that they have received funds from the Public Health Agency of Canada to develop, disseminate, and evaluate educational resources on family violence, including child maltreatment.

The remaining authors declare that the research was conducted in the absence of any commercial or financial relationships that could be construed as a potential conflict of interest.
